# A New Approach to 3D Modeling of Inhomogeneous Populations of Viral Regulatory RNA

**DOI:** 10.3390/v12101108

**Published:** 2020-09-29

**Authors:** Patrick S. Osmer, Gatikrushna Singh, Kathleen Boris-Lawrie

**Affiliations:** 1 Department of Astronomy, The Ohio State University, Columbus, OH 43210, USA; osmer.1@osu.edu; 2 Department of Veterinary and Biomedical Sciences, University of Minnesota, Saint Paul, MN 55108, USA; gsingh@umn.edu

**Keywords:** 5′-cap, centroid, complex 5′-untranslated region, intramolecular folding, medoid, retrovirus, RNA structure ensemble, supercomputer cluster

## Abstract

Tertiary structure (3D) is the physical context of RNA regulatory activity. Retroviruses are RNA viruses that replicate through the proviral DNA intermediate transcribed by hosts. Proviral transcripts form inhomogeneous populations due to variable structural ensembles of overlapping regulatory RNA motifs in the 5′-untranslated region (UTR), which drive RNAs to be spliced or translated, and/or dimerized and packaged into virions. Genetic studies and structural techniques have provided fundamental input constraints to begin predicting HIV 3D conformations in silico. Using SimRNA and sets of experimentally-determined input constraints of HIV^NL4-3^ trans-activation responsive sequence (TAR) and pairings of unique-5′ (U5) with dimerization (DIS) or AUG motifs, we calculated a series of 3D models that differ in proximity of 5′-Cap and the junction of TAR and PolyA helices; configuration of primer binding site (PBS)-segment; and two host cofactors binding sites. Input constraints on U5-AUG pairings were most compatible with intramolecular folding of 5′-UTR motifs in energetic minima. Introducing theoretical constraints predicted metastable PolyA region drives orientation of 5′-Cap with TAR, U5 and PBS-segment helices. SimRNA and the workflow developed herein provides viable options to predict 3D conformations of inhomogeneous populations of large RNAs that have been intractable to conventional ensemble methods.

## 1. Introduction

Intramolecular folding of RNA secondary structures drives tertiary conformation of RNA molecules [1,2,3]. The HIV-1 5′-untranslated region (HIV 5′-UTR) contains overlapping structural motifs that regulate early and late replication events [4,5,6,7,8,9,10]. Within the 5′-UTR, unspliced proviral transcripts engage host ribonucleoproteins (RNPs) that catalyze splicing or translation to virion proteins, or viral nucleocapsid RNPs drive pairs of RNA molecules into diploid genomic RNPs (gRNPs) to be packaged into virions. Rather than be trapped in a single multifunctional conformation, HIV 5′-UTRs exist as inhomogeneous populations transiting metastable structures that are impractical to capture experimentally [11].

Ample secondary structure information is available on individual segments of the HIV 5′-UTR that are necessary for dimerization and packaging of diploid gRNA into virions [6,9,12,13,14,15,16,17,18,19,20]. The dimer-prone secondary structure exhibits pairing of primary sequences of the unique 5′ region (U5) and AUG regions (U5-AUG model) [4,8]. U5-AUG pairing orients the dimer initiation sequence (DIS) for intermolecular dimerization and the core encapsidation signal (CES) for nucleocapsid binding, and packaging of the diploid genomic ribonucleoprotein into virions [8].

In-solution studies have established the dimer-prone secondary structure (U5-AUG model) exists in thermodynamic equilibrium with U5 pairing DIS (U5-DIS model) [4]. U5-DIS pairings reorient metastable AUG and CES sequences into branched multiple hairpins that characterize the monomer 5′-UTR (non-dimer prone) [4,8]. Nucleotide pairings favoring the dimer-prone 5′-UTR conformation or monomer conformation have been examined in-solution. Substitutions in DIS or destabilizing U5-AUG pairings diminish formation of dimers in synthetic RNAs and or RNA preparations from cells and virions [21].

Select base substitutions that destabilize U5-AUG and unpair adjacent PolyA nts significantly upregulate the HIV RNA translation rate in infected lymphocytes, demonstrating monomer structure significantly affects activity [22]. Unpaired PolyA nts have been shown to influence the structural topology of the 5′-cap site and modulate engagement by host cap-binding proteins [6,23]. The phylogenetic conservation of PolyA indicates that experimentally changing these nt–nt pairings may promote maximum viral translation efficiency, but does not necessarily maintain optimal efficiency of other structural motifs within the viral 5′-UTR [22].

RNA molecules fold in a hierarchical pathway, primary sequence folding into two-dimensional (2D) multi-helical loops, bulges and stems that undergo intramolecular folding by three-dimensional (3D) interactions [1,24,25,26,27]. The 3D interactions are composed of pseudoknots, non-canonical base pairings, and single or unstacked base pairs, non-canonical (not A–U, G–C, and G–U) base pairs, pseudoknots, triplet and G-quadraplex interactions. Importantly, biological functionality of RNA in 3D requires metastable regions to be energetically compatible with helices trapped in energetic minima [1,3,24].

3D models can provide a testing ground to interrogate base substitutions, including those that destabilized U5-AUG and PolyA stem to significantly upregulate HIV translation rate [28]. Current computational methods have the ability to predict 3D RNA conformations within small parameter space (<160 nt) or within larger molecules given biologically-determined input constraints [3,29,30]. Tertiary structure prediction tools, such as SimRNA have been compared and validated in the RNA Puzzles experiments [31,32,33]. SimRNA begins with known structural constraints in PDB format that have been derived from chemical and enzymatic mapping, crystallography, NMR, small angle X-ray scattering (SAXS), or Förster resonance energy transfer (FRET) and optimally, are validated by mutagenesis and biological assays. Structural information validated by genetic studies has been published of progressively larger fragments of HIV 5′-UTRs, providing a solid foundation of 2D input constraints for 3D modeling [4,6,9,12,13,14,15,16,17,18,19,20,34,35]. 3D models have the potential to begin to guide experiments characterizing contiguous HIV 5′-UTRs in large 3D ensembles.

Herein we used SimRNA to perform simulations of the contiguous HIV 5′-UTR starting from published 2D pairings of TAR, PolyA, CES and U5-DIS (monomer) or dimer-prone U5-AUG (herein designated dimer). SimRNA evaluated the positive predictive value (PPV) of the input constraints and the sensitivity of output models. Input constraints were varied based on experimentally determined metastable U5 pairings and theoretical variables. Perspective on the capability of SimRNA to simulate HIV RNA interactions with centroid secondary structure, or no secondary structure constraint was also developed.

## 2. Materials and Methods

The HIV-1^NL4-3^ 5′-UTR primary sequence was downloaded from NCBI https://www.ncbi.nlm.nih.gov/nuccore/AF324493 4 September 2015 and the 5′-UTR starting with two 5′ guanosine residues (Cap + G-356 nt) was studied using the SimRNA program [32,33]. The input to SimRNA was primary sequence and experimentally-determined 2D structural constraints provided in the dot-bracket language [36]. Variables in the input constraints were designated Monomer (U5-DIS model), Dimer (dimer-prone, U5-AUG model) and centroid WT with/out PolyA unpaired [4,37]. The specific parameters for the SimRNA calculations were 30 million iterations with 10 replica exchanges, for a total of 300 million iterations. Each SimRNA run required on order 60 h of clock time on the Pitzer Cluster at The Ohio State Supercomputer Center. SimRNA provided outputs in dot-bracket language, 3D images and PDB files. Agreement between input restraints and output base pairings were benchmarked for sensitivity and positive-predictive values. Input and Output nt–nt pairings were compared in the dot-bracket language, converted to Circle plots by CircleCompare (https://rna.urmc.rochester.edu/RNAstructureWeb/Servers/CircleCompare.html, 1 December 2018) and traditional 2D sequence models were drawn in Adobe Photoshop 6. Visual Molecular Dynamics software package (VMD 1.9.3) [38] produced static views and movies of the Output models.

## 3. Results

### 3.1. SimRNA Evaluated 3D Properties of HIV^NL4-3^ 5′-UTR Beginning with 5′-Capped-Guanosine

**Figure 1:**
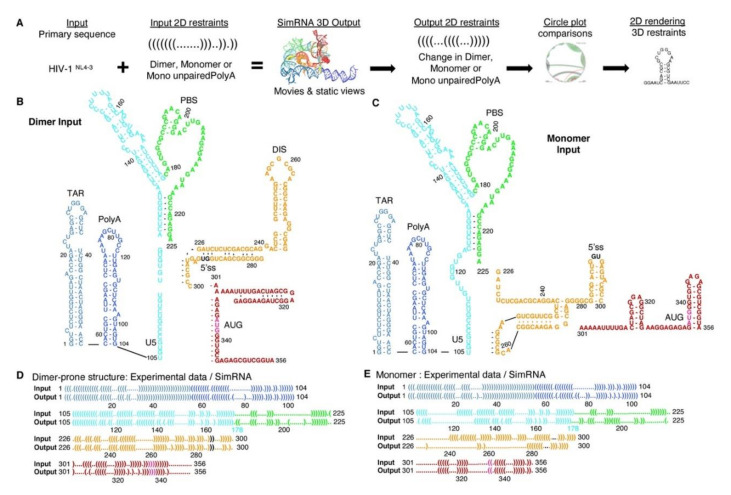
Approach to generate tertiary structure models of the HIV^NL4-3^ 5′-UTR. (**A**) Workflow used to process the tertiary structure predictions by SimRNA from experimentally determined models expressed in the Vienna dot-bracket language. (**B**) Input Dimer secondary structure input to SimRNA. (**C**) Comparison of the Input and Output structures in the Vienna dot-bracket language. (**D**) Monomer secondary structure input to SimRNA. (**E**) Comparison of the experimental Input constraints and SimRNA Output models in the Vienna dot-bracket language. Label colors designate: TAR, light blue; PolyA stem, navy; U5 stem, cyan; PBS, green; DIS and 3-way junction, orange; AUG stem, red; 5′-splice site (5′ss), black; gag start codon, magenta.

All simulations used the same HIV^NL4-3^ primary sequence of 356 nt commencing with guanosine residues (5′-Cap + G). The workflow relied upon experimentally determined 5′-UTR conformers (Figure 1A). Perspective on the capability of SimRNA to simulate HIV RNA was developed by comparing in silico constraints of centroid 2D or no secondary structure (noSS) inputs. The dimer-prone (herein designated Dimer) and Monomer 2D models (Figure 1B,C) were previously determined by analysis of HIV RNA fragments [4,6,9,37]. The color scheme for each 5′-UTR conformation visualized TAR, light blue; PolyA stem, navy; U5 stem, cyan; PBS, green; DIS stem, orange (Figure 1B).

Input constraints were provided to SimRNA in Vienna dot-bracket language showing unpaired bases (dot) in relation to paired bases (brackets) (Figure 1D). SimRNA output the medoid of the three most populated clusters of nt–nt pairings having the lowest 2% of free energy (Cluster 1, 2 or 3). Medoid of the largest cluster is the most representative tertiary conformation engendered by the input restraints (Cluster 1) and was displayed using VMD software and dot-bracket language according to the workflow (Figure 1A).

SimRNA Output from the Dimer and Monomer Input restraints generated similar numbers of objects in the top 3 clusters, as expected for identical primary sequences (Table S1). The dot-bracket language readily visualized the position of each difference between the input and output (Figure 1D). Dimer exceeded Monomer in the number of nt–nt pairings in common between the Input restraints and Output medoid (95% and 74%, respectively) (sensitivity, Table 1). Dimer Output also exceeded Monomer in the number of nt–nt pairings retained in 3D Output model that are in the Input experimentally-determined constraints (PPV, Table 1). These measurements indicated the dimer-prone U5-AUG pairings were energetically compatible with intramolecular folding of metastable regions between helices trapped in energetic minima.

### 3.2. Dimer Output 3D Model Predicted TAR-PolyA-U5 Converge near the 5′-Cap

**Figure 2:**
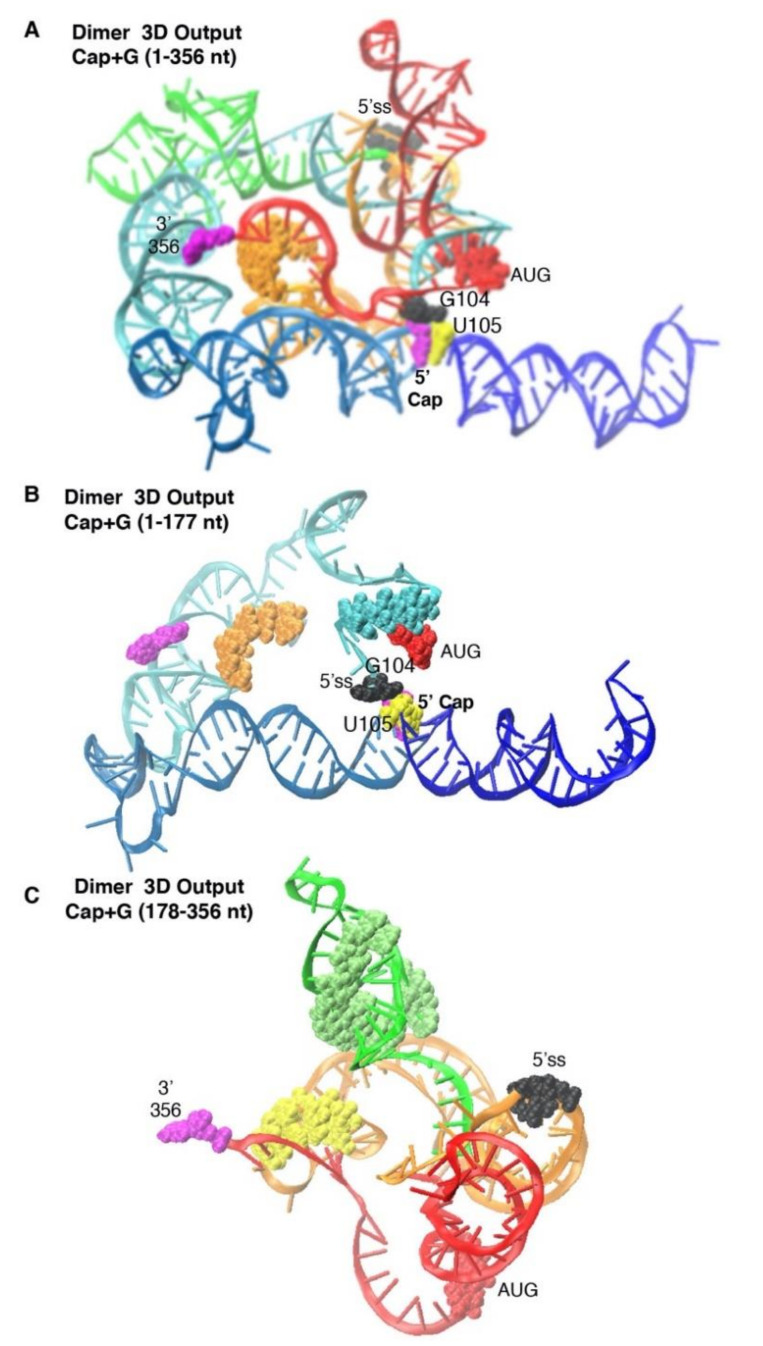
Dimer 3D model identified by SimRNA. (**A**) Most representative model of Dimer-prone 5′-UTR. (**B**) Focus on 5′-Cap + 1–177. (**C**) Focus on nt 178–356. Label colors designate: TAR, light blue; PolyA stem, navy; U5 stem, cyan; PBS, green; DIS and three-way junction, orange; 5′-Cap and 3′ terminus, magenta atoms; G104, black atoms; U105, yellow atoms; AUG stem, red atoms. 5′-splice site (5′ss), black atoms; DIS atoms, orange (**A**,**B**), yellow; gag start codon, red atoms.

The Dimer 3D tertiary prediction placed TAR (light blue) and PolyA (navy) helices stacking on one another (Figure 2A; Movie 1). These results agree with published results of chemical mapping [8,10,39,40] and SAXS [34,35]. The 5′-Cap (magenta space-filling atom) lay at the junction of TAR and PolyA helices and the base of the U5 stem near G104-U105. In Figure 2B, the 3D Output focused on the U5 nt (cyan atoms) pairing with AUG (red atoms), matching the input constraint. 5′-Cap was stacked on G104-U105 (white and yellow atoms) at the junction of TAR and PolyA, and AUG was in close proximity, albeit paired with U5.

**Figure 3:**
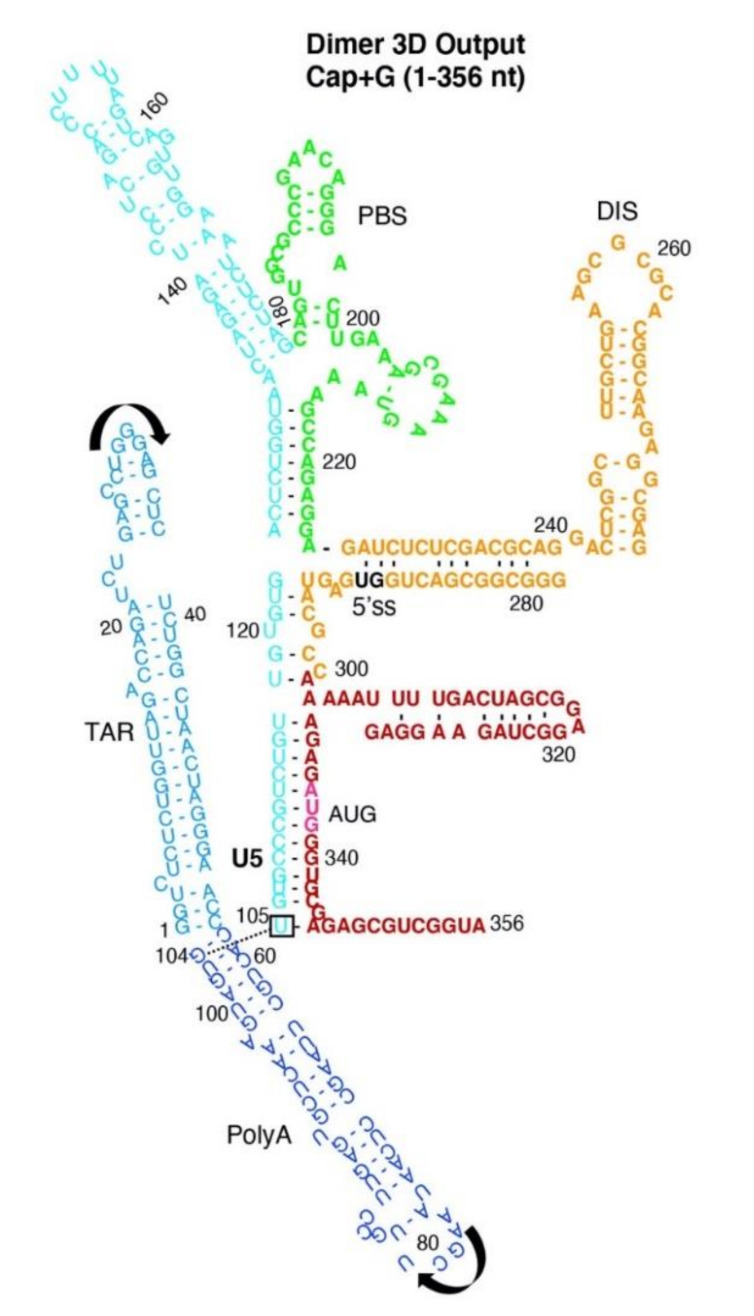
2D rendering of SimRNA 3D Dimer Model. Arrows represent the orientation of TAR and PolyA helices. Box and dashed line indicates stacking of G104-U105. Label colors: TAR, light blue; PolyA, navy; U5 stem, cyan; PBS-segment, green; DIS stem, orange; AUG stem, red; 5′-splice site (5′ss), black; AUG translation start codon, magenta.

In Figure 2C, the 3D Output focused on the green PBS-segment (nt 180–225) and that DIS was surface-exposed (see yellow atoms). The 5′-splice site (ss) lay between helices formed by nts 300–330 of CES (Movie 1) [37]. To emphasize the differences in nt–nt pairings between input and output, Dimer Output pairings were displayed in a Circle plot and transcribed into the traditional secondary structure format (Figure 3). Since 96% of the Input constraints maintained (Table 1), the ensemble exhibited minimal change from the Input nt–nt pairings provided for Dimer (Figure 2B). The 3D model predicted stacking of G104-U105 that had not been apparent in 2D Input constraints. The model predicted the PBS-segment (nt 134–224) containing the double-stranded primer activation signal (PAS) [41] and the tRNA-like element (TLE) at the apex of helices formed of nt 134–179 [42] and, at the base of the helices, the double-stranded RNA binding site of DHX9/RNA helicase A (RHA) [28].

### 3.3. Monomer Output 3D Model Altered Accessibility of 5′-Cap and 5′-ss and Reoriented U5 Stem

**Figure 4:**
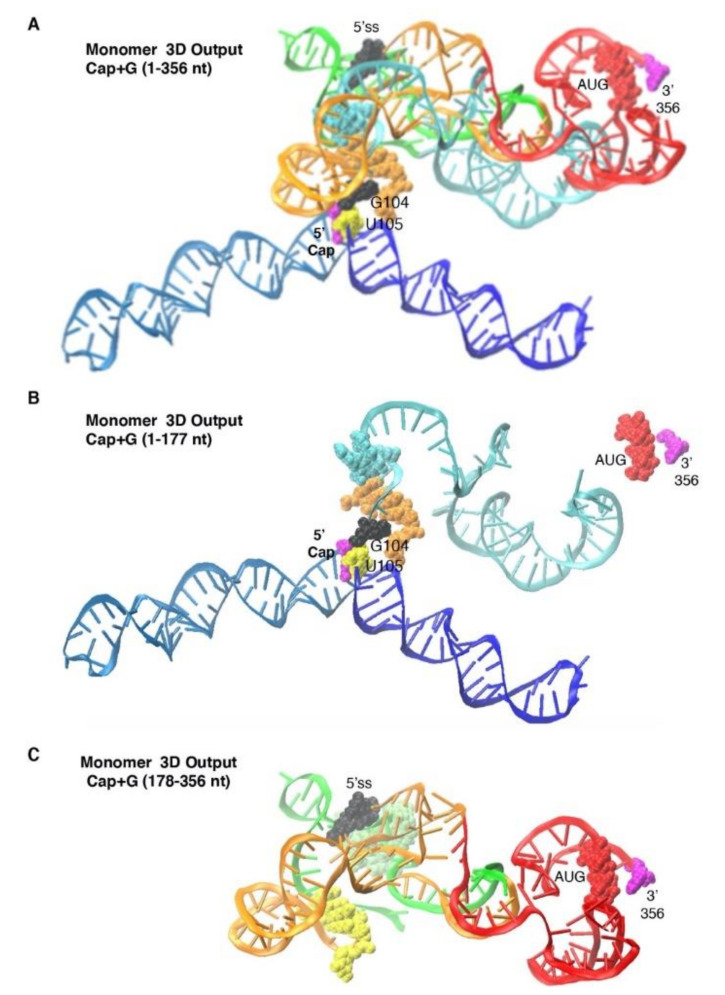
Monomer 3D model identified by SimRNA. (**A**) Most representative model of Monomer-prone 5′-UTR. (**B**) Focus on nt 1–177. (**C**) Focus on nt 178–356. Label colors designate: TAR, light blue; PolyA stem, navy; U5 stem, cyan; PBS, green; DIS stem and three-way junction, orange; 5′-Cap and 3′ terminus, magenta; G104, black; U105, yellow; AUG stem, red. 5′-splice site (5′ss), black; DIS, orange (**A**,**B**), yellow (**C**); gag start codon, red.

The Monomer 3D model identified TAR and PolyA helices, but in a different orientation than Dimer (Figure 4A; Movie 2). Whereas Dimer modeled coaxial arrangement of the TAR-PolyA helices, Monomer modeled V-shape due to differences in stacking interactions. Figure 4B focused on the close proximity of 5′-Cap, G104, U105 and DIS (orange atoms). Helices formed of U5-DIS pairs and downstream orange nt (e.g., 256–260) showed the 5′-Cap intercalated between these helices (compare Figure 4A,B; Movie 2). Figure 4C focused on the 5′ss at the apex of a stem loop. Gag AUG was paired close to the 3′-terminus of the 5′-UTR. The models depict TAR-PolyA-U5 stems intersect around the 5′-Cap (Figure 4A).

Notably, only 74% of the Input nt–nt pairings maintained in Monomer Output (Table 1). The lower positive predictive value of Monomer versus Dimer (74% versus 87%, Table 1) suggested U5-DIS helices significantly changed intramolecular folding of 5-UTR relative to Dimer.

**Figure 5:**
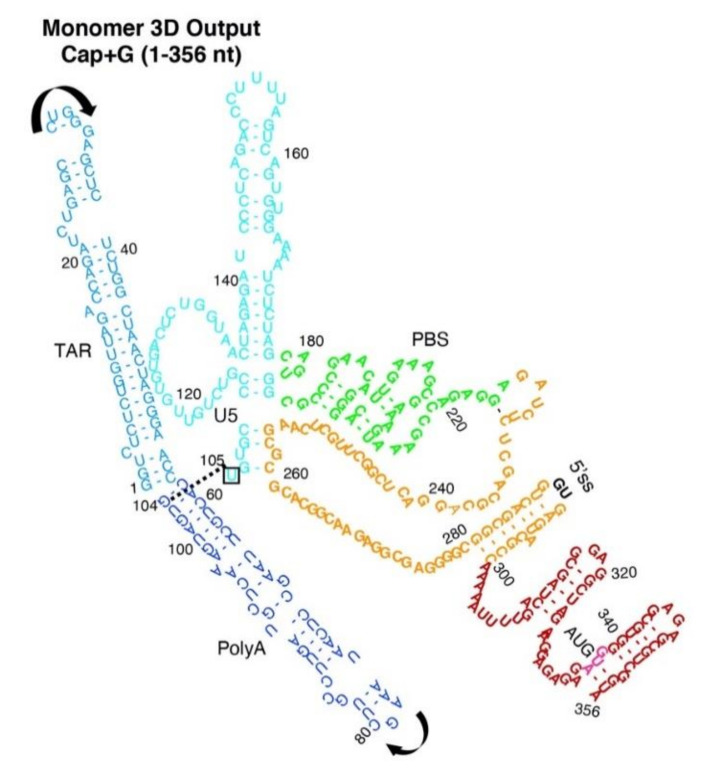
2D rendering of SimRNA 3D Monomer model. Arrows represent the direction of TAR or PolyA helices. Label colors designate: TAR, light blue; PolyA, navy; U5 stem, cyan; PBS-segment, green; DIS stem, orange; AUG stem, red; 5′-splice site (5′ss), black; nt of the 3-way junction, orange. Space filling atoms: 5′-Cap, white; G104, yellow; U105, black; AUG translation start codon, red or magenta.

Circle plots compared the different nt pairings between Monomer Input restraints and Monomer Output (Supplementary Figure S1) and were used to generate the traditional secondary structure drawing (Figure 5). The nt–nt pairings of TAR and PolyA maintained in the 3D Output model, but new pairings encompassed the U5 stem (cyan) through the PBS-segment (green) (compare Figure 5 with Figure 1C), consistent with the long-distance interaction model of Huthoff and Berkhout [8]. The 3D Monomer model eliminated the nt-nt pairings of PAS [41], TLE [42] and the binding site of DHX9/RHA [28]. We concluded tertiary interactions drove rearrangement of metastable Monomer Input restraints in silico. The Output model predicted three elements important to reverse transcription were incompatible with U5-DIS pairings.

### 3.4. The Unpairing of PolyA Nts Reduced Local Energy Minima in the Thermodynamic Equilibrium to Monomer Tertiary Structure

Unpaired residues modulate local energy minima in the thermodynamic equilibrium between structural intermediates [43,44]. Biological evidence has unequivocally demonstrated the nt–nt pairings observed in TAR are essential for HIV transcriptional trans-activation by Tat [45,46,47,48], whereas the nt–nt pairings of the PolyA stem are less stringently required [49]. To test Input constraints without PolyA pairing, we provided SimRNA the identical Monomer Input restraints, except residues 59–103 were unpaired (Mono PolyA unpaired).

SimRNA calculated 300 million iterations of the input restraints comparing Monomer and Mono PolyA unpaired. Output benchmarks improved for Mono PolyA unpaired, indicating metastable PolyA region favored intramolecular folding of the nt–nt pairings in energetic minima (Table 1). The percentage of pairings maintained between the Output and Input restraints was 87%, compared to 74% for Monomer (PPV, Table 1). Notably, PolyA pairings (*n* = 17) were completely restored in the top clusters of SimRNA models (Clusters 1 and 2, Figure S2).

**Figure 6:**
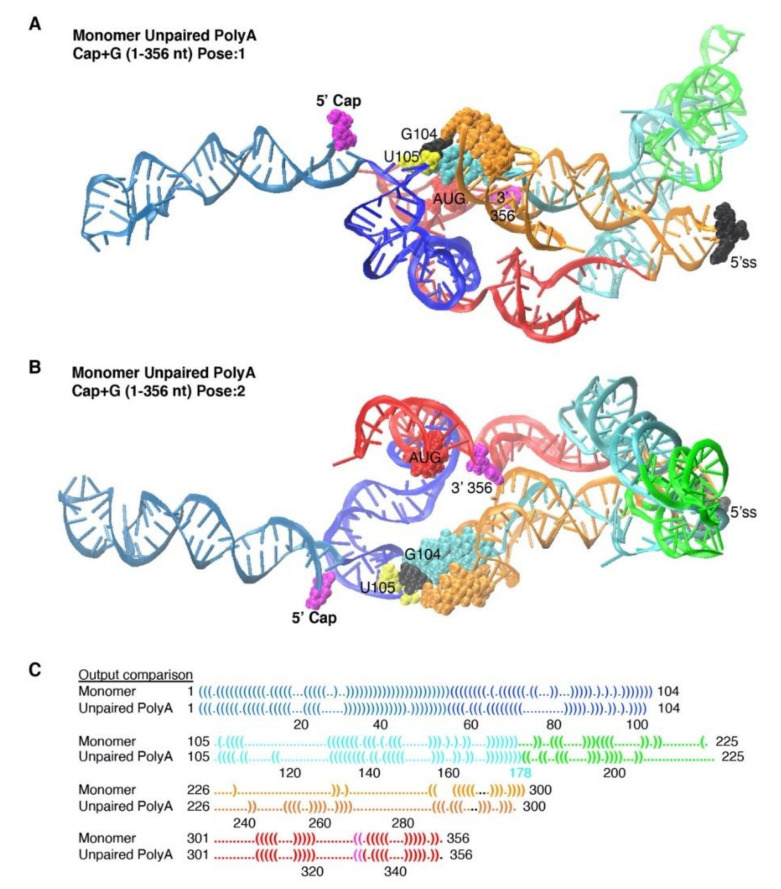
The SimRNA simulation of Monomer is changed by unpairing PolyA. (**B**) Two poses of the most representative SimRNA model of Monomer unpaired PolyA. (**C**) Vienna dot-bracket language comparison of differences between Input and Output constraints. The colored font designates: TAR, light blue; PolyA, navy; U5 stem, cyan; PBS-segment, green; DIS stem, orange; AUG stem, red; 5′-splice site (5′ss), black; nt of the 3-way junction, orange. Space filling atoms: 5′-Cap, white; G104, yellow, U105, black, AUG translation start codon, red (**A**,**B**) or magenta (**C**).

Inspection of the 3D Output identified the expected helical structure of TAR (Figure 6A,B) and U5 residues (105–110) paired with DIS residues (256–259) (Figure 6A, see cyan and orange space-filling atoms) (Movie 3). The results suggest metastable PolyA nts tethered TAR and U5-DIS helices trapped in energetic minima.

The metastable PolyA region changed proximity of 5′-Cap to G104 and U105 at the base of the U5 stem (Figure 6A,B; Movie 3) and reoriented AUG near to U5, rather than near to PBS as observed in Monomer (Figure 6A,B compared with Figure 4B). The 5′ss remained at the apex of a stem loop, yet was not intercalated between the PBS and residues of the 3-way junction, as observed in Monomer (Figure 6A,B compared with Figure 4C).

Approximately 80% of the nt–nt pairings were in common with Monomer and all of the differences were downstream from PolyA stem (Figure S2). We concluded the ensemble space of 5′-UTR conformation was reduced by PolyA nt pairings. These observations agree with the prior finding that unpaired residues play an important, passive role in HIV 5′-UTR secondary structure [7,50]. In sum, stacking of 5′-Cap with G104-U105 favored by input restraints pairing the PolyA nts.

### 3.5. TAR Input 2D Restraints Significantly Influence SimRNA Models

To add perspective on the power of laboratory versus computationally-derived constraints useful for SimRNA modeling, we ran two additional models, the first using the secondary structure of the centroid of the HIV ensemble as input and the second using no 2D constraint, only the primary sequence as input data (noSS). The centroid is an ab initio thermodynamic average of HIV^NL4-3^ 2D ensemble (herein designated Centroid WT) [36,51,52]. Whereas the thermodynamic average of 2D RNA structures integrates the minimum free energy (MFE) of canonical base pairings (G-C, A-U, G-U) and folding constraints of nearest neighbor bases within short RNA strands [51], advances in dynamic programming algorithms take this approach a step further by monitoring ensembles of metastable structures [52]. This dynamic approach samples nt–nt pairings, calculating probabilities of concurrent sub-structures from the ensemble of metastable structures and structures trapped in energetic minima. Convergence on probabilistic structures is required to produce the predominant stems, bulges, internal and multi-helical loops and provide the most representative model, or centroid.

**Figure 7:**
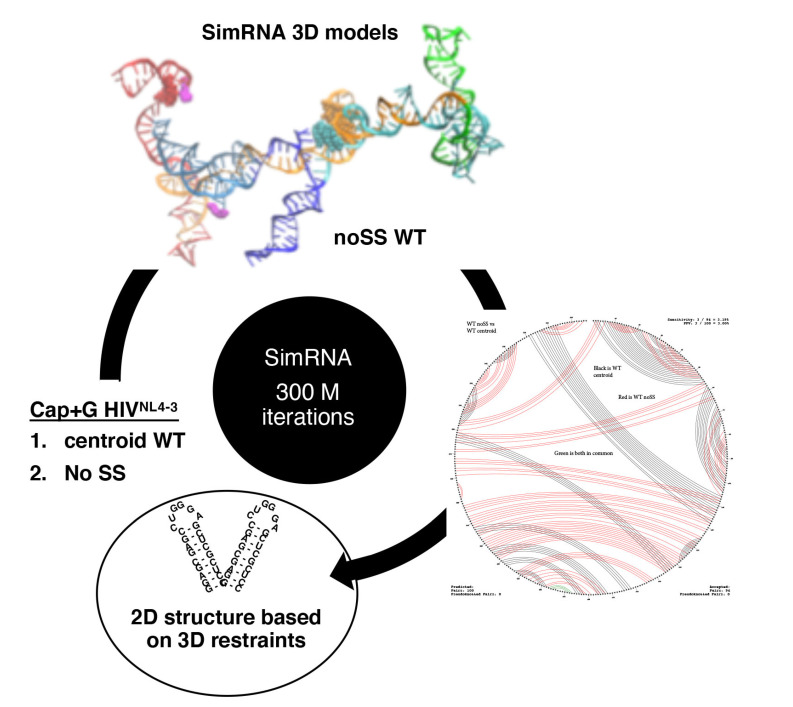
Approach to generate tertiary structure of HIV^NL4-3^ 5′-UTR using theoretical Input constraints. Workflow presents two sources of theoretical Input restraints provided to SimRNA to predict 3D models of the HIV 5-UTR in the course of 300 million iterations on the Pitzer Supercomputer Cluster. The most representative 3D models were backed into Vienna dot-bracket language and 2D features were analyzed using CircleCompare and used to render 2D comparison with the input 2D model. The 3D model and Circleplot present the SimRNA simulation of HIV 5′-UTR with no secondary structure restraint (no SS). Label colors designate: TAR, light blue; PolyA stem, navy; U5 stem, cyan; PBS stem, green; DIS stem, orange; AUG stem, red. Atoms: 5′-Cap and 3′ nt, magenta; U5, cyan; DIS, orange; 5′-splice site (5′ss), black; gag start codon, red.

Centroid WT Input constraints did not demonstrate input constraints additional to the experimentally-determined Monomer and Dimer Input 2D constraints (Figure S3A,B). Centroid WT secondary structure prediction mimicked the U5-AUG pairings observed for Dimer. Next, the computationally-derived Centroid WT input was processed 300 million iterations by SimRNA and the workflow processed results of the theoretical input constraints (Figure 7). Centroid WT Input pairings maintained and the top output cluster had 12 additional pairings (Figure S2C).

**Figure 8:**
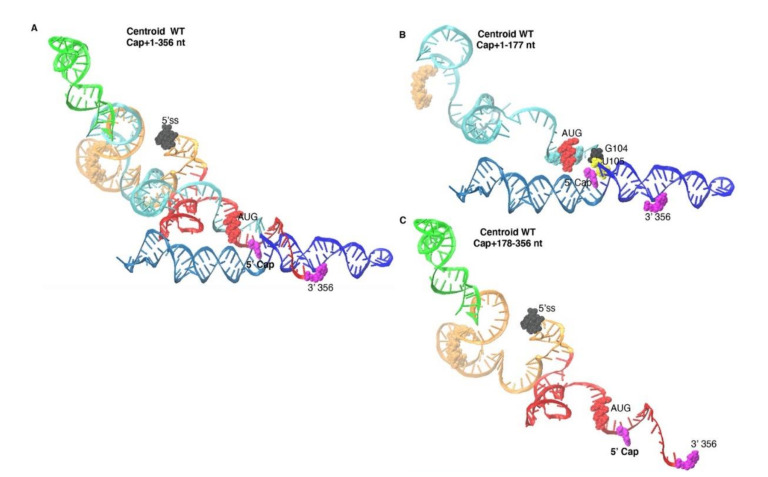
Output of Centroid WT predicted U5-AUG pairing and apical position of 5′ss. (**A**) 3D model predicted by SimRNA based on Centroid WT theoretical input constraints. (**B**) Focus on 5′-Cap + 1–177. (**C**) Focus on nt 178–356. Colors designate: TAR, light blue; PolyA, navy; U5 stem, cyan; PBS-segment, green; DIS stem and 3-way junction, orange; AUG stem, red; 5′-splice site (5′ss), black. Space filling atoms: 5′-Cap and 3′ terminus, magenta; G104, yellow, U105, black, AUG translation start codon, red.

**Figure 9:**
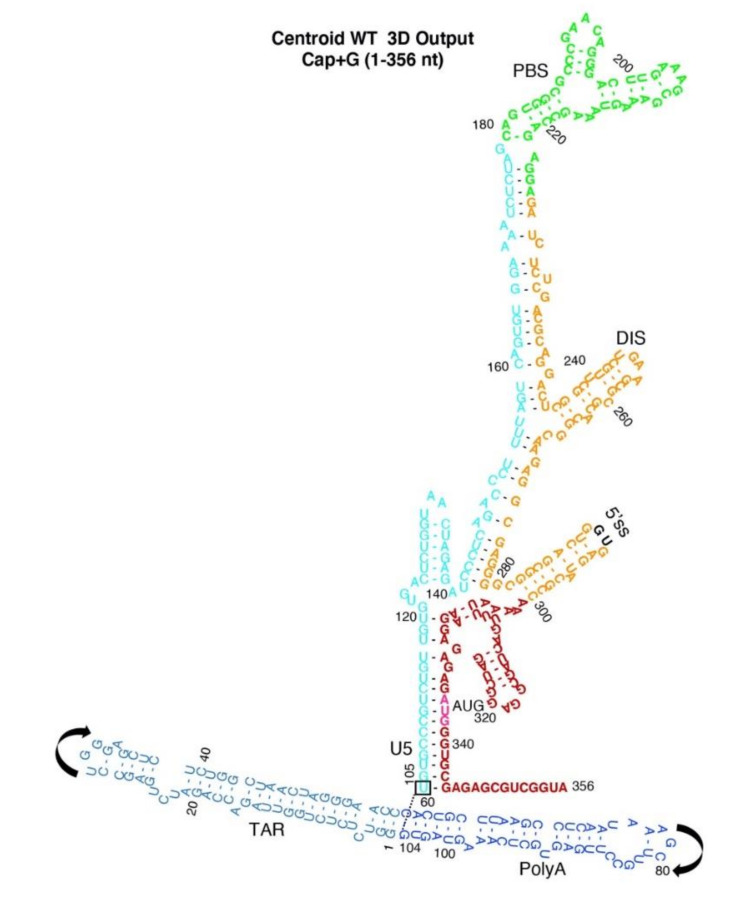
2D rendering of Centroid WT Output. Arrows represent the direction of TAR or PolyA helices. The colored font designates: TAR, light blue; PolyA, navy; U5 stem, cyan; PBS-segment, green; DIS stem, orange; AUG stem, red; 5′-splice site (5′ss), black; nt of the 3-way junction, orange. Space filling atoms: 5′-Cap, white; G104, yellow, U105, black, AUG translation start codon, red or magenta.

Centroid WT predicted TAR and PolyA arranged coaxial helices and base stacking interaction between Cap, G104-U105 (Figure 8A,B compared with Figure 4A,B). Unlike Dimer, the 5′ss was positioned at the apex of the stem loop formed by nt 280–300, as observed in Monomer (Figure 8A,C compared with Figure 4A,C). The U5 stem was an extended helix (nt 141–177 and 225–280) topped with PBS (green) (Figure 8A,C and Figure 9), similar to structural intermediates of HIV 5′-UTR dimerization [43,44,46,50].

The Output from Centroid WT recapitulated TAR and PolyA stems and pairing of U5 nts 134–140 (Figure 8 and Figure 9). As summarized in Table 2, 60% of the identified pairings were maintained in Dimer and Monomer (Table 2), indicating overlap in the parameter spaces. The 3D simulations of Centroid WT Input restraints predicted thermodynamic stability of U5-AUG pairings, similar to Dimer, and apical position of the 5′ss that is similar to Monomer.

Output ensembles of centroid WT and noSS had similar numbers of total objects as Dimer and Monomer (Table S1). However, the Output from noSS Input constraints recapitulated only 3% of the pairings within centroid WT (Table 2). These results indicated the energetic cost of TAR was excessive within the parameter space of 300 million iterations. Given noSS Input constraints, SimRNA sampled metastable structures without constraint on TAR, a condition comparable to basal HIV transcription that is Tat/TAR-independent independent. The results suggested the TAR helix significantly influences the thermodynamic equilibrium of the first ~100 nt of the 5′-UTR.

## 4. Discussion

In the course of 300 million iterations of dynamic assemblies, SimRNA evaluated the 3D parameter space of the 5′-UTR beginning with experimentally-determined 2D constraints, theoretical constraints on PolyA nt pairing, and the secondary structure centroid prediction of its whole Boltzmann ensemble. SimRNA predictions suggest TAR helical stem and metastable PolyA region are fundamental input constraints determining the proximity of 5′-Cap to the junction of TAR and PolyA helices and the base of the U5 stem near G104-U105. The SimRNA 3D modeling agreed in principle with recent NMR results that PolyA nt pairing/unpairing propagates structural rearrangements throughout the 5′-UTR, perturbing the orientation of 5′-Cap, TAR and U5 residues, [6,53].

The metastable nt pairings in U5 and PBS-segment likely contribute to the alternate functionalities of the 5′-UTR in cellulo. Destabilized PBS-segment reduces affinity for DHX9/RHA and diminishes processivity of reverse transcriptase and virus infectivity [28,54,55]. Destabilized A59-U103 pairing significantly increase Gag translation rate in cells [22] and increase biochemical affinity of the HIVMAL 5′-Cap for host eIF4E [6]. The HIV late RNAs have been shown to specifically retain nuclear cap-binding complex (CBC) in polysomes and virions, instead of exchanging to eIF4E [56]. Recently, polysomal CBC RNPs were shown to activate eIF4E-independent translation of stress response proteins, overcoming AP-1 protein translation attenuation by mTOR inhibition [57]. Structural malleability near the HIV 5′-Cap may be beneficial to regulate cap-exchange and antagonize antiviral mTOR inhibition.

Tertiary modeling of polypeptide conformations has been integral to elucidating the functional mechanisms of enzymes, chaperones, many structural proteins and receptor-ligand interactions and for in silico modeling of small molecule therapeutics. Tertiary modeling of RNA structures has robust potential to guide the rational design of antiviral therapeutics [58,59,60,61]. Supportive evidence that alternative nt–nt pairings propagate structural changes throughout the HIV-1 leader RNA includes TAR-binding compounds exert structural effects outside TAR [62,63] and small molecule binding within the CES reduce virus titer [64]. Our results suggest programs like SimRNA can help to begin to predict RNA structure-function relationships testable in structural studies and cell-based studies [3].

## 5. Conclusions

By incorporating experimental and theoretical constraints, and performing 300 million iterations of dynamic ensembles, the SimRNA software package was used to simulate tertiary conformations of the HIV^NL4-3^ 5′-UTR. The Output models predicted tertiary interactions in context of U5-AUG pairings placed G104-U105 stacking on 5′-Cap and maintained helices of the PBS-segment previously identified as PAS, TLE [41,42] and the double-stranded RNA binding site for DHX9/RHA [28]. Alternative U5 pairing with DIS reduced SimRNA agreement of input constraints with tertiary models. In conclusion, secondary structures that support early and late events in HIV replication divergently effect the 5′-UTR 3D models identified by the SimRNA algorithm. The simulation workflow developed herein provides a viable approach to model inhomogeneous large RNA populations.

## Figures and Tables

**Table 1:** SimRNA parsed nt–nt pairings of the experimentally-determined input restraints.

5′ Input Restraints ^a^	Sensitivity ^b^	PPV ^c^
Dimer	106/111 (96%)	105/111 (95%)
Monomer	73/99 (74%)	73/99 (74%)
Mono Unpaired PolyA ^d^	77/99 (78%)	77/89 (87%)
WT centroid	94/94 (100%)	94/123 (76%)

^a^ HIV ^NL4-3^ 5′-untranslated sequence beginning with 5′-Cap-guanosine. ^b^ Sensitivity, pairs common to 2D Input constraints and 3D Output model. ^c^ PPV, positive predictive value. Pairs retained in 3D Output model that are in the Input structure. ^d^ Monomer Unpaired nt +59 through +103. The nt–nt pairings of Input and Output were enumerated and compared by the CircleCompare program (https://rna.urmc.rochester.edu/RNAstructureWeb/Servers/CircleCompare.html). The percentage of nt pairs in the Input that were identified by SimRNA indicated sensitivity. The percentage of nt pairs identified by SimRNA that were in the Input structure measured positive predictive value (PPV).

**Table 2:** Input restraints strongly influence positive predictive value of SimRNA.

HIV ^NL4-3^ 5′-UTR 2D Restraints ^a^	Sensitivity ^b^	PPV ^c^
Input	Versus Centroid WT Output	
Monomer	54/94 (58%)	54/99 (55%)
Dimer	58/94 (62%)	58/111 (52%)
noSS WT ^d^	3/100 (3%)	3/94 (3%)

^a^ HIV ^NL4-3^ 5′-untranslated sequence beginning with 5′-Cap-guanosine. ^b^ Sensitivity, pairs identified in both Input and SimRNA Output. ^c^ PPV, positive predictive value, pairs identified by SimRNA that are in the Input structure. The nt–nt pairings of SimRNA models were enumerated and compared by the CircleCompare program (https://rna.urmc.rochester.edu/RNAstructureWeb/Servers/CircleCompare.html) 12 December 2018. The percentage of nt pairs in each Input model that were identified by SimRNA in centroid WT model indicated sensitivity. The percentage of nt pairs identified in each Input that were in centroid WT model measured positive predictive value (PPV). ^d^ noSS, no secondary structure input constraint.

## Supplementary Materials

The following are available online at https://www.mdpi.com/1999-4915/12/10/1108/s1, Figure S1: Circle plots of Input and Ouput nt-nt pairings identified for Dimer-prone and Monomer HIVNL4-3. Figure S2: Circle plots comparing 3D output of Monomer with the top 3 clusters of Monomer UnpairedPolyA (cluster 1, 2, 3). Figure S3. Comparison of Input restraints of centroid WT to Dimer or Monomer. Table S1. Number of objects in the top 3 ensembles of objects having the lowest 2% of free energy, Video S1: SimRNA Output 3D Model of Dimer-prone 5′-UTR. Video S2: SimRNA Output 3D Model of Monomer 5′-UTR. Video S1: SimRNA Output 3D Model of Mono PolyA unpaired.
