# Supplementary material for: A New Approach to 3D Modeling of Inhomogeneous Populations of Viral Regulatory RNA
Source: Viruses. 2020 Sep 29;12(10):1108. doi: 10.3390/v12101108 (PMC7650772; doi:10.3390/v12101108)
Supplement: Supplementary file 1 [file viruses-12-01108-s001.zip › viruses-871804-suppr1.pdf]

viruses

Supplementary Materials: A New Approach to 3D Modeling of Inhomogeneous Populations of Viral Regulatory RNA

Patrick S. Osmer, **Gatikrushna Singh** and **Kathleen Boris-Lawrie**

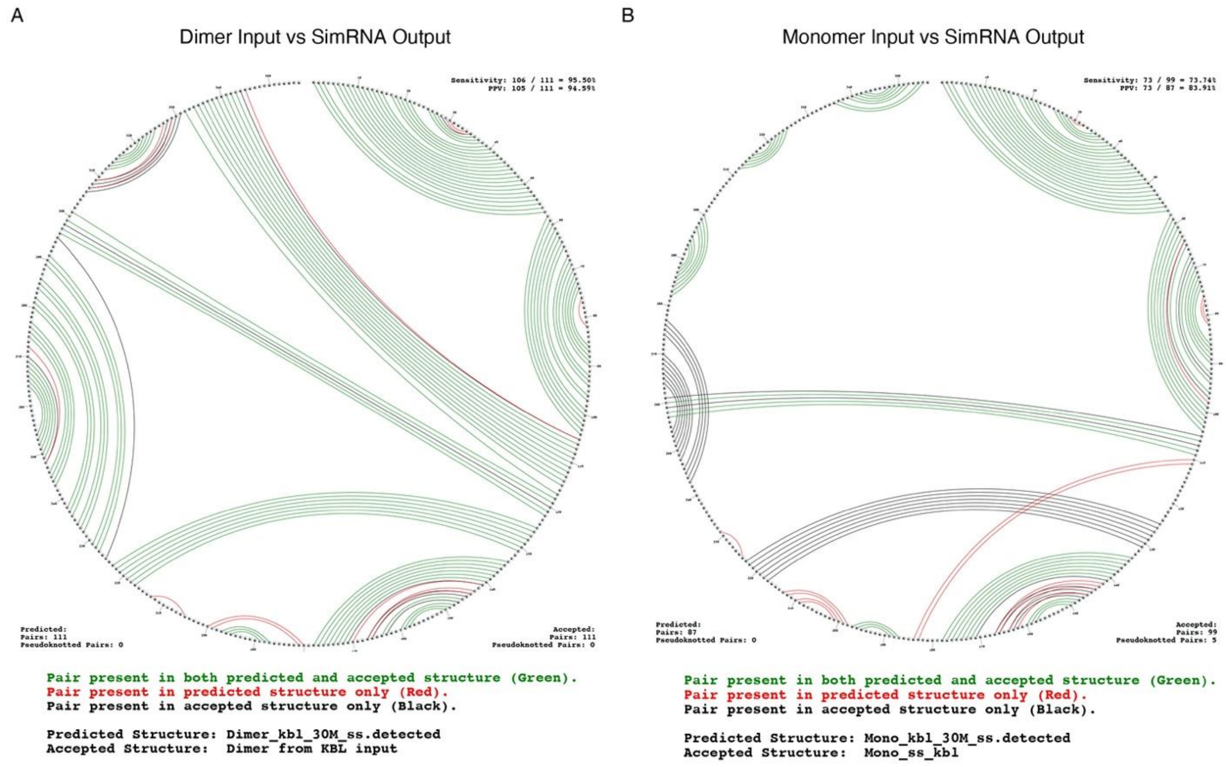

Figure S1. Circle plots showing the primary sequence of HIV^{NL4-3} (edges labeled in 10 nt increments) and lines showing the nt-nt pairings predicted by SimRNA (Output model). (A) Dimer Input restraints compared to the Dimer Output model; (B) Monomer Input restraints compared to the Dimer Output model. Green, pairings in common for SimRNA Output and the respective Input restraints. Red, pairings exclusive to Dimer Output. Black, pairings exclusive to Input restraints.

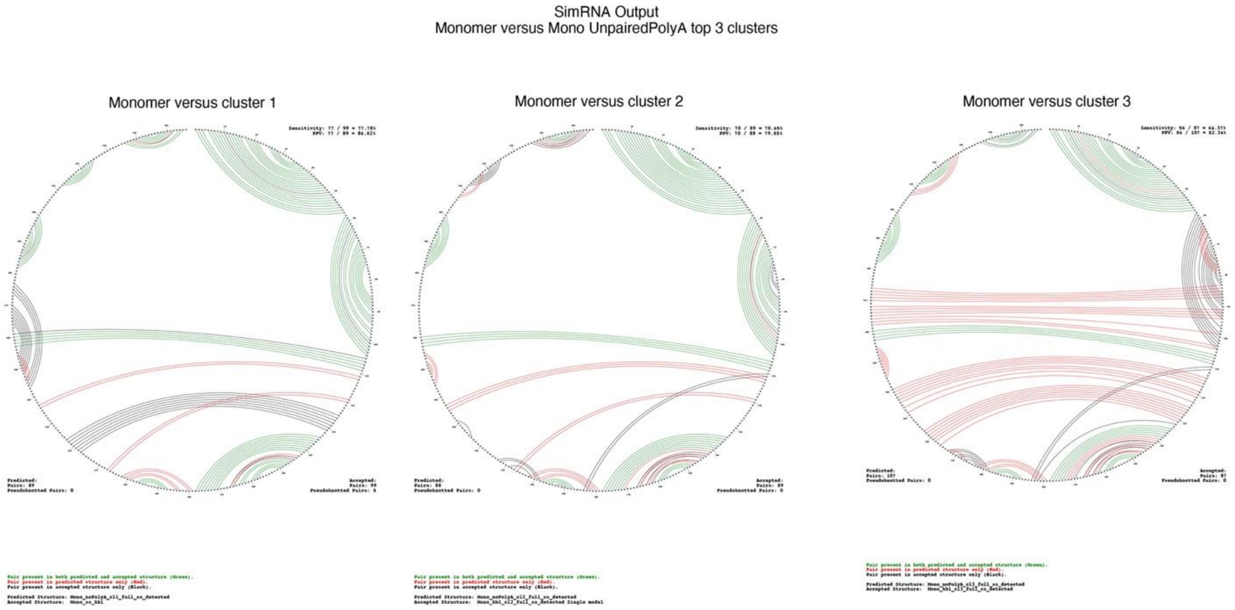

Figure S2. Circle plots comparing 3D output of Monomer with the top 3 clusters of Monomer UnpairedPolyA (cluster 1, 2, 3). Green, pairings present in both Monomer Output and cluster Output. Red lines, pairings exclusive to Monomer Output. Black, pairings exclusive to cluster Output.

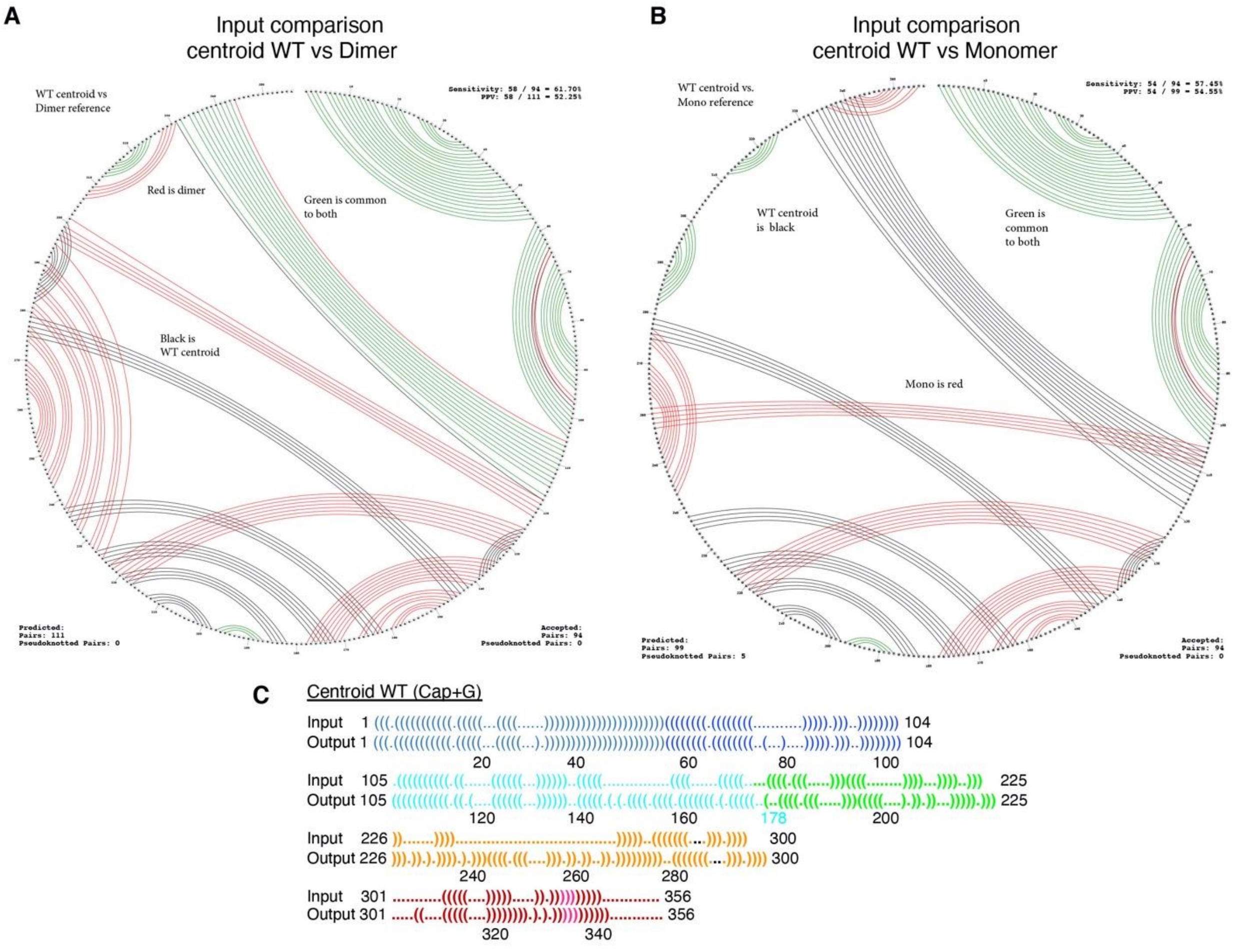

Figure S3. Comparison of Input restraints of centroid WT to Dimer or Monomer. SimRNA predicted nt-nt pairings visualized using CircleCompare plots and translated to Vienna dot-bracket language. A) Green, pairings in common for centroid WT and Dimer. Red, pairings exclusive to Dimer. Black, exclusive to centroid WT. Green, pairings in centroid WT and Dimer or Monomer. Red, pairings

exclusive to Dimer. Black, exclusive to centroid WT. B) Green, pairings in common for centroid WT and Monomer. Red, pairings exclusive to Monomer. Black, exclusive to centroid WT. Green, pairings in common for centroid WT and Monomer. Red, pairings exclusive to Monomer. Black, exclusive to centroid WT. C) Dot-bracket files comparing centroid WT Input restraints with 3D Output show high similarity except nt 240-265. Color scheme as in Fig. 1.

Table S1. Number of objects in the top 3 ensembles of objects having the lowest 2% of free energy.

**Number of models in top three clusters of each simulation** [a]				
**Input restraint**	**Dimer**	**Monomer**	**Mono Unpaired PolyA**	**centroid WT**
1	206	196	182	240
2	160	150	160	154
3	142	142	146	126
Total	508	488	488	520

[a] The clustering algorithm identifies members of all clusters that have RMSD structural differences within the threshold value of ~10 Angstroms.
